# Toll-Like Receptor 2 Expression as a New Hallmark of Advanced Endometriosis

**DOI:** 10.3390/cells9081813

**Published:** 2020-07-30

**Authors:** Małgorzata Sobstyl, Paulina Niedźwiedzka-Rystwej, Ewelina Grywalska, Izabela Korona-Głowniak, Anna Sobstyl, Wiesława Bednarek, Jacek Roliński

**Affiliations:** 1 Chair and Department of Gynecology and Gynecological Endocrinology, Medical University of Lublin, 20-037 Lublin, Poland; malgorzata.sobstyl@umlub.pl; 2 Institute of Biology, University of Szczecin, 71-412 Szczecin, Poland; 3 Department of Clinical Immunology and Immunotherapy, Medical University of Lublin, 20-093 Lublin, Poland; sobma@poczta.onet.pl (A.S.); jacek.rolinski@gmail.com (J.R.); 4 Department of Pharmaceutical Microbiology, Medical University of Lublin, 20-093 Lublin, Poland; iza.glowniak@umlub.pl; 5 I Chair and Department of Gynecological Oncology and Gynecology, Medical University of Lublin, 20-081 Lublin, Poland; wieslawa.bednarek@umlub.pl

**Keywords:** endometriosis, dendritic cells, innate immunity, lymphocytes, monocytes, Toll-like receptor 2

## Abstract

Recent evidence suggests that immunological aspects play a pivotal role in this disorder. Toll-like receptor 2 (TLR2) is crucial in recognizing microbial infections and mediating innate immune response. The objective of our study was to rate with flow cytometry the levels of several subsets of dendritic cells, monocytes, and basic peripheral blood lymphocytes expressing TLR2, aiming at the determination of a possible correlation between the expression of TLR2 and the clinical outcomes of endometriosis in 40 patients and 40 age-matched healthy women. Our study showed the importance of TLR2 expression, mainly on myeloid dendritic cells (mDCs) and B cells in patients with endometriosis. Both mDCs BDCA1+CD19-TLR2+ and B lymphocytes CD19+TLR-2+ proved useful in the differentiation of affected individuals with stages 3–4 of the disease (area under the receiver operating characteristic curve /AUC/ = 0.96, *p* < 0.0001 for mDCs; AUC = 0.78, *p* = 0.0001 for B lymphocytes), and those presenting adhesion (AUC = 0.92, *p* < 0.0001 for mDCs; AUC = 0.82, *p* < 0.0001 for B lymphocytes) or infertility (AUC = 0.83, *p* < 0.0001 for mDCs; AUC = 0.73, *p* = 0.006 for B lymphocytes). Our findings suggest that the levels of TLR2-expressing cells, particularly mDCs and B lymphocytes, may be an effective biomarker of endometriosis, because the disease currently lacks clinically useful noninvasive biomarkers enabling early and cost-effective diagnosis.

## 1. Introduction

Endometriosis is an estrogen-dependent inflammatory disease of chronic character associated with variable degrees of pelvic pain and infertility [1]. It is assumed that nearly 10% of women are affected with this difficult condition [2]. A diagnosis of endometriosis can be suspected based on the presence of pelvic and non-gynecological cyclical symptoms, but they are not disease-specific [3]. To confirm the diagnosis a combination of laparoscopic observations and histological examination of tissue samples collected during laparoscopy or other invasive procedures is considered the gold standard [3]. A few promising putative biomarkers of endometriosis have been reported, including those associated with nerve fiber growth and cell cycle control in the endometrium [4], or blood-derived proteomic or metabolomic fingerprints [5]. Nevertheless, the availability of clinically useful noninvasive biomarkers, as well as the combination of non-invasive tests for the diagnosis of endometriosis is very limited [5,6,7], and none of them is currently recommended in clinical practice [3]. 

The pathogenesis of this disorder is not fully understood, and it seems to be very complex and unclear; nevertheless, recent evidence suggests that immunological aspects play a pivotal role in endometriosis [8]. Without doubt, changes in cells of the immune system in endometriosis are noted, mainly among dendritic cells, monocytes/macrophages, natural killer cells, and T and B cells [8,9,10,11]. Potentially available from peripheral blood, several immunological factors, including cytokines, antibodies, or complement components, have been investigated as putative noninvasive biomarkers of endometriosis [5,6]. However, none of them was proven useful in clinical settings [5,6].

Dendritic cells (DCs) are an incredible connection between innate and adaptive immunity [12,13]. As a main subset of antigen-presenting cells, they are typically differentiated to conventional—myeloid DC (cDCs, mDCs) and plasmacytoid DC (pDCs) [12]. cDCs are characterized by the expression of cluster of differentiation (CD) 45 marker, main histocompatibility complex (MHC) II, and CD11c, and the lack of T cell, NK cell, B cell, granulocyte, and erythrocyte lineage markers, and their role is wide ranging—from inducing innate immunity cascade to priming naïve T cells [12]. pDCs express low levels of MHCII and CD11c, but they have a strong ability to produce high levels of interferon (IFN) type I, and their role in antiviral immunity, T responses, and autoimmune disease is prominent [13]. Monocyte-derived peritoneal DCs from endometriosis samples were shown to overexpress mannose receptor on their surface, which was associated with increased phagocytosis of dead endometrial cells, contributing to the development of endometriosis [14]. The findings from a murine model on the role of DCs in endometriosis are contradictory, with some reports showing enhancement of endometriotic lesion growth and angiogenesis upon the addition of DCs [15,16], whereas others demonstrated that DCs impair lesion development by stimulating T-cell activity [17].

The role of monocytes/macrophages is primary either as reside macrophages (after reaching peripheral tissues) or antigen-presenting cells [8,18]. Among monocytes, two main subsets are enumerated, depending on the expression of surface markers—classical monocytes CD14+CD16- and nonclassical CD14+CD16+ [19]. Classical monocytes express CCR2 receptor for chemokine CCL2 and CD64, CD93, CD163, and GM-CSF (granulocyte-macrophage colony stimulating factor), whereas nonclassical monocytes express CX3CR1 and HLA-DR, and the expression on CD64 is relatively low [20]. In endometriosis, macrophages are responsible for early immune escape of endometriotic cells [21], and they contribute to the establishment and growth of endometriotic lesions by promoting the production of inflammatory cytokines [18].

Within the family of T cells, several subsets are known to be crucial in different diseases, pathogen-associated and autoimmunity [22,23]. Mainly, T helper cells (Th) and T regulatory cells (Tregs) are involved, while Th1 are known for their pro-inflammatory actions, and Th2 are rather anti-inflammatory [22]. To maintain pivotal immune homeostasis, Tregs with the CD4+CD25+Foxp3+ phenotype are important, as well as producing IL-10 and TGF-β, together with several anti-inflammatory cytokines [23]. Both Th and Treg cells were shown to be involved in the pathogenesis of endometriosis [24,25]. The percentage of Th1 lymphocytes was significantly higher in endometriosis tissue than in endometrial cells [24]. The same pattern was observed for peripheral Th1 cells: its proportion was significantly higher in patients with endometriosis than in those without endometriosis [24]. Patients with ovarian endometriosis had a higher percentage of CD25^high^ FOXP3^+^ Treg cells in the peripheral blood than the control group [25]. These findings highlight the potential of T lymphocytes as noninvasive biomarkers of endometriosis.

The diversity of B cells is not extensive, and these cells are known to be responsible for antibody production via the generation of memory B cells or antibody-forming plasma cells [26]. A pivotal characteristic of B cells is the expression of CD19 receptor, essential for the activation of B cells [26]. The role of antibodies in endometriosis was extensively investigated, but a possible autoimmune disease background is still debated [8]. The incidence of antibodies, including anti-endometrial antibodies, is higher in women with endometriosis than in those without endometriosis [27]. However, no such differences were observed for levels of B cells [28].

Finally, none of the immune system cells would operate successfully, if not for the proper signaling of the immunological pathways. One of the most significant signaling pathways is via TLR (Toll-like receptors) - a significant subset of PRR (pathogen recognition receptors), recognizing a wide range of PAMPs (pathogen-associated molecular patterns) [29,30]. In the TLR family, several types of ligands are recognized to be of bacterial, viral, and other origin, depending on the type of TLR. TLR2 is known to be mainly antibacterial, involved in the recognition of bacterial lipopeptide, and functioning in cooperation with TLR1, TLR4, and TLR6, by forming functional heteromeric complexes [1,31,32]. 

Taking the above into consideration, it can be stated that, apart from a wide range of TLRs, mainly TLR2 roles in bacterial, viral, and fungal infections [33], together with immunological role as a crucial signaling element of innate immunity [34], TLR2 may have a pivotal role in the pathogenesis of patients with endometriosis. Being expressed on many immunological cells, TLR2 also recognizes different endogenous types of alarmins [35], meaning that not only PAMPs, but also danger-associated molecular patterns (DAMPs) are in control of these receptors, maintaining the homeostasis of the organism on the appropriate level [36]. 

The aim of the studies was to evaluate the levels of several subsets of immunological cells (dendritic cells, monocytes, and basic peripheral blood lymphocytes) expressing TLR2, in order to determine a possible correlation between the expression of TLR2 and the clinical outcomes of endometriosis.

## 2. Materials and Methods 

### 2.1. Collection of Peripheral Blood

Peripheral blood was collected from the ulnar vein of previously untreated patients newly diagnosed with endometriosis and healthy volunteers using sterile, EDTA-coated blood collection tubes in an amount of 5 mL (for flow cytometric analysis of basic lymphocytes subsets) (S-Monovette^®^, SARSTEDT, Aktiengesellschaft and Co., Numbrecht, Germany) and using heparin-coated blood collection tubes in an amount of 2 mL (for flow cytometric analysis of monocytes and DCs) (Blood Gas Monovette^®^, Luer, Lithium Heparin calcium-balanced, SARSTEDT, Aktiengesellschaft and Co., Numbrecht, German). As a part of a routine procedure in our hospital, serum levels of Cancer Antigen 125 (CA-125) and Human Epididymis Protein 4 (HE4) antigens were determined. These well-established ovarian cancer biomarkers [37] are also proposed as endometriosis markers [38].

### 2.2. Flow Cytometry and Sample Preparation: Monocytes and Dendritic Cells

The percentage of monocytes and peripheral blood dendritic cells expressing TLR2 was measured using flow cytometry. The preparation of the material for sampling was the following: 100-µL samples of whole blood from each participant were incubated for 20 min in the dark with pairs of fluorochrome-conjugated monoclonal antibodies against the following markers: BDCA-1 (CD1c) FITC (Biolegend, San Diego, CA, USA)/ anti- CD3, CD14, CD16, CD19, CD20, CD56 Lineage Cocktail (Lin) Pacific Blue/ TLR2 PE (Biolegend, San Diego, CA, USA ); BDCA-2 FITC (Biolegend, San Diego, CA, USA) / CD123 Pe-Cy7 (Biolegend, San Diego, CA, USA )/ CD45 V450 (Biolegend, San Diego, CA, USA)/ TLR2 PE (Biolegend, San Diego, CA, USA ); and CD14 FITC (BD Biosciences, San Jose, CA, USA) / CD16 V450 (BD Biosciences, San Jose, CA, USA) / HLA-DR Pe-Cy7 (BD Biosciences, San Jose, CA, USA)/ TLR2 PE (Biolegend, San Diego, CA, USA ). Afterwards, the samples were treated with lysis buffer (Lysing Buffer, BD Pharm Lyse, San Jose, CA, USA) and washed in PBS solution (Sigma-Aldrich, Saint Louis, MO, USA). The samples were collected using a Cytoflex LX (Beckman Coulter, CA, USA) and analyzed using the Kaluza Analysis program. The collected data were evaluated using spot plots. Myeloid dendritic cells were defined as BDCA1+Lin- cells, and plasmacytoid dendritic cells were defined as BDCA2+CD123+ cells. Classical monocytes were defined as CD14+CD16- cells, and non-classical monocytes were defined as CD14+CD16+ cells. To improve monocyte purity, an additional gating step with HLA-DR was used. Isotype controls (Biolegend, San Diego, CA, USA) were used to determine unspecific binding, and sample analyses are shown in Supplementary Figure S1, and a sample analysis of TLR-2+ monocytes, myeloid, and plasmacytoid dendritic cells is shown in Supplementary Figure S2. 

### 2.3. Flow Cytometry and Sample Preparation: Lymphocytes, Natural Killer, and Natural Killer T-Like Cells

Analogically, as in Section 2.2, the percentage of peripheral blood lymphocytes expressing TLR2 was cytometrically measured. The amount of blood for sampling was 50 µL, and the following antibodies were used: CD19 FITC /CD3 PE / (BD Biosciences, San Jose, CA, USA), CD4 FITC/ CD8 PE / CD3 PerCp (BD Biosciences, San Jose, CA, USA), CD3 FITC/CD16 PE/CD56PE (BD Biosciences, San Jose, CA, USA), TLR 2 PE / CD4FITC (BD Biosciences, San Jose, CA, USA), TLR2PE / CD8FITC (BD Biosciences, San Jose, CA, USA), and TLR2PE / CD19FITC (BD Biosciences, San Jose, CA, USA). Next, the samples were treated with lysis buffer (Lysing Buffer, BD Pharm Lyse, San Jose, CA, USA) then washed in PBS solution (Sigma-Aldrich, Saint Louis, MO, USA). The samples were collected using a Cytoflex LX (Beckman Coulter, CA, USA) and analyzed using the Kaluza Analysis program. The collected data were evaluated using spot plots. Isotype controls (Biolegend, San Diego, CA, USA) were used to determine unspecific binding, and sample analyses are shown in Supplementary Figure S3, and a sample analysis of TLR-2+ T CD4+, T CD8+, and B CD19+ lymphocytes is shown in Supplementary Figure S4.

Additionally, we measured the percentages of T helper (CD3+CD4+), T cytotoxic (CD3+CD8+), B lymphocytes (CD3−CD19+), NK (CD3−CD16+CD56+), and NKT-like cells (CD3+CD16+CD56+). The target cell population was determined by using the forward and lateral dispersions (single-color dispersion vs. lateral dispersion) and a two-color fluorescence plot. The relative percentage of cells expressing the surface markers was quantified by placing gates around the individual populations. The results are presented as the percentage of CD45+ cells. The percentage of cells was calculated by comparison with the control. Isotype-matched, directly conjugated murine FITC IgG1 κ isotype control and PE IgG1 κ isotype control were used to determine the background signal, to exclude contamination and cell aggregates.

### 2.4. Statistical Analysis

The statistical analysis was carried out with Tibco Statistica 13.3 (StatSoft, Palo Alto, CA, USA). Normal distribution of continuous variables was tested using the Shapiro–Wilk test. The values of the parameters were presented as arithmetic means and their standard deviations (SD) for normally distributed data and as medians, minimum, and maximum values for non-normal data. Student’s t-test was used for independent variables and the Mann–Whitney U-test as an intergroup comparison component. Kruskal–Wallis ANOVA and multiple comparisons of mean ranks (as post-hoc analysis) were applied for the analysis of differences between more than two groups. The power and direction of association between pairs of continuous variables (studied groups) were determined using Spearman’s coefficient of rank correlation. The distributions of discrete variables in groups were compared with the Pearson’s chi-square test or Fisher’s exact test. Additionally, the diagnostic effectiveness of the laboratory test was determined using receiver operating characteristic (ROC) curves for parameters related to the different groups of patients. Areas under the ROC curves (AUCs) were calculated for each parameter and compared. The error was set at 5% and significance at *p*-value <0.05.

## 3. Results

### 3.1. Characteristics of the Patients and Controls 

The study group included 40 prospective patients of the I Chair and Department of Gynecological Oncology and Gynecology of the Medical University of Lublin with confirmed diagnosis of endometriosis. Blood samples were obtained from previously untreated women with suspected endometriosis one day before surgery. Only patients who had endometriosis confirmed intraoperatively or in the histopathological examination following the surgery were included in the study. The control group comprised 40 age-matched healthy female volunteers reporting neither gynecological nor non-gynecological cyclical symptoms indicative of endometriosis, and whose health status was confirmed based on the results of routine diagnostic examinations conducted during control check-up visits to a gynecologist.

The criteria for exclusion from the study were as follows: taking medications affecting immune reactions, hormonal preparations, signs of infection visible at least four weeks prior to the study, blood transfusion, and the presence of autoimmune disease, pregnancy, lactation, cancer, or allergies. The study protocol was approved by the Local Bioethical Committee of the Medical University of Lublin (approval no. KE-0254/251/2014, 27 September 2018), prior to the beginning of the study. All patients gave written informed consent to use their blood samples for scientific purposes. Endometriosis was diagnosed based on standard diagnostic criteria and confirmed postoperatively according to European Society of Human Reproduction and Embryology guidelines [3]. The stage of endometriosis was evaluated according to the revised American Society of Reproductive Medicine (rASRM) classification [39].

The characteristics of the patients and controls, including the serum levels of CA-125 and HE4 antigens, are presented in Table 1. 

### 3.2. Dendritic Cells, Monocytes, and Basic Peripheral Blood Lymphocyte Subsets and Expression of TLR2 Antigen in Healthy Individuals and Patients with Endometriosis

**Figure 1:**
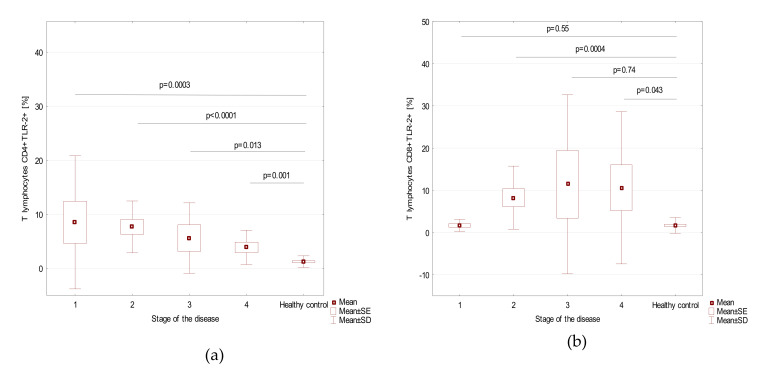

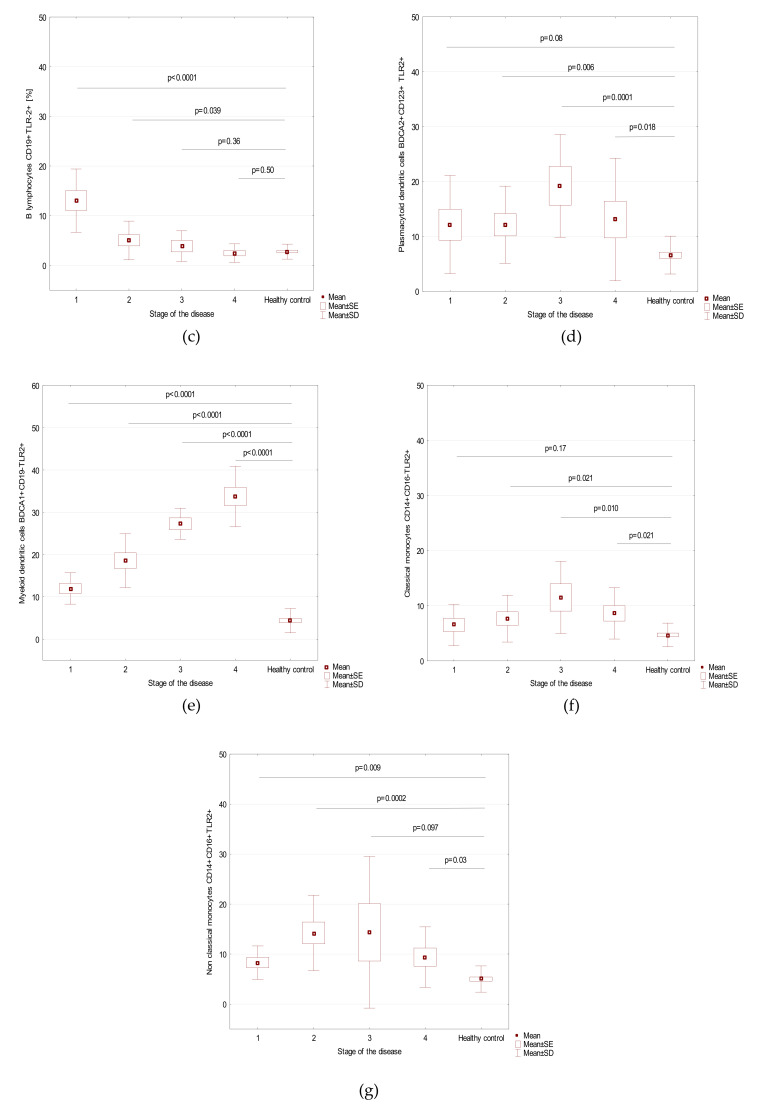
TLR2+ biomarkers in endometriosis patients with different stages of disease and in the control group. (**a**) T lymphocytes CD4+TLR-2+ [%]; (**b**) T lymphocytes CD8+TLR-2+ [%]; (**c**) B lymphocytes CD19+TLR-2+ [%]; (**d**) Plasmacytoid dendritic cells BDCA2+CD123+ TLR2+ [%]; (**e**) Myeloid dendritic cells BDCA1+CD19-TLR2+ [%]; (**f**) Classical monocytes CD14+CD16-TLR2+ [%]; (**g**) Non-classical monocytes CD14+CD16+TLR2+ [%]. Mean values are marked as squares, means ± standard errors as boxes, and mean ± standard deviation as whiskers.

Table 2 shows the content of the dendritic cells, monocytes, and basic peripheral blood lymphocytes subsets and the percentage of these cell subsets expressing TLR2 antigen in the patients and controls, and Table 3, in patients with stages 1–4 of endometriosis. Figure 1 shows the prevalence of different subsets of TLR2+ cells in patients with stages 1–4 of endometriosis and in the control group. We found a statistically significant correlation between the frequencies of T CD4+TLR2+ lymphocytes and HE4 serum concentration (*r* = 0.395; *p* = 0.0112), as presented in Supplementary Figure S5.

### 3.3. Receiver Operating Characteristic (ROC) Curve Analysis to Determine the Diagnostic Accuracy of TLR2 Expression on Myeloid Dendritic Cells, Plasmacytoid Dendritic Cells, Monocytes, and T and B Lymphocytes in Patients with Endometriosis vs. Controls

**Figure 2:**
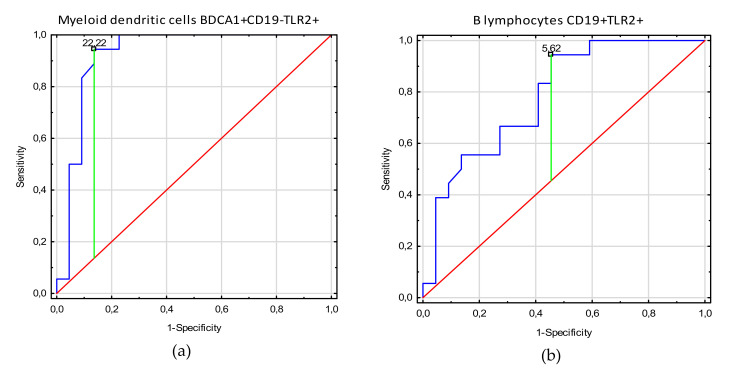
Receiver operating curve (ROC) analysis to determine diagnostic accuracy in differentiation of patients with endometriosis stages 3–4 and with endometriosis stages 1–2: (**a**) frequencies of myeloid dendritic cells BDCA1+CD19-TLR2+ [%]; (**b**) frequencies of B lymphocytes CD19+TLR2+ [%].

**Figure 3:**
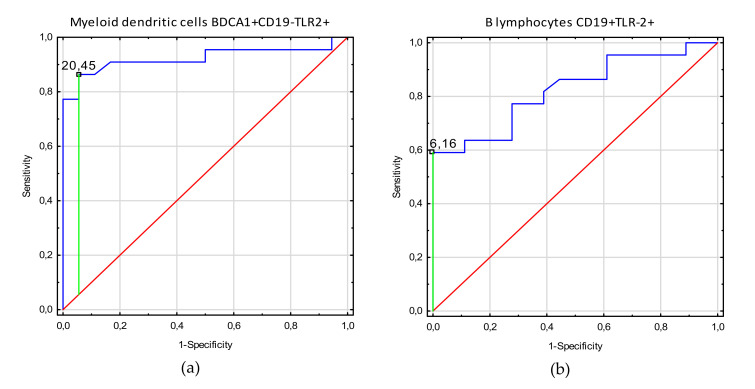

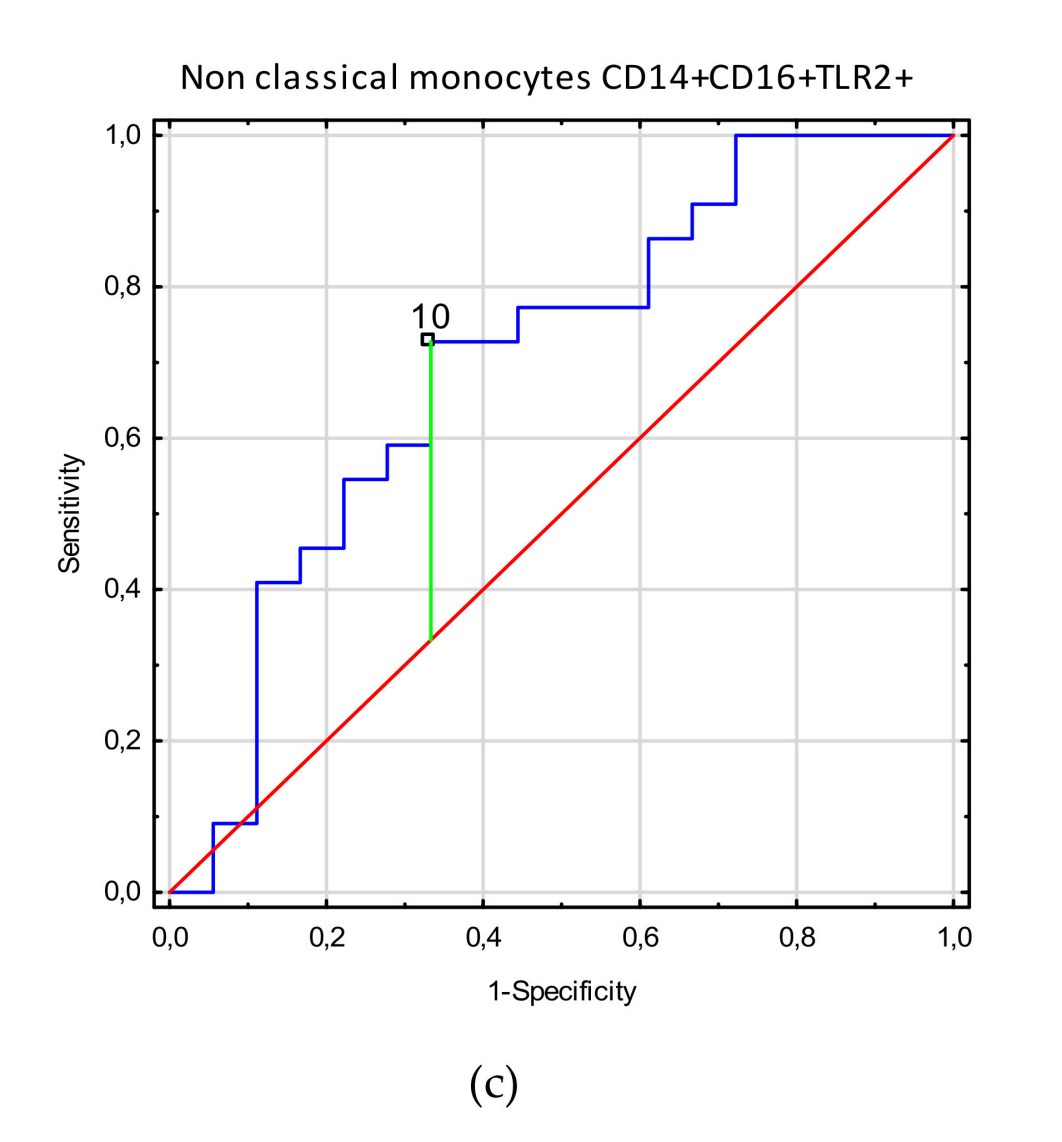
Receiver operating curve (ROC) analysis to determine diagnostic accuracy in differentiation of endometriosis patients with and without adhesions: (**a**) frequencies of myeloid dendritic cells BDCA1+CD19-TLR2+ [%]; (**b**) frequencies of B lymphocytes CD19+TLR2+ [%]; (**c**) frequencies of non-classical monocytes CD14+CD16+TLR2+ [%].

**Figure 4:**
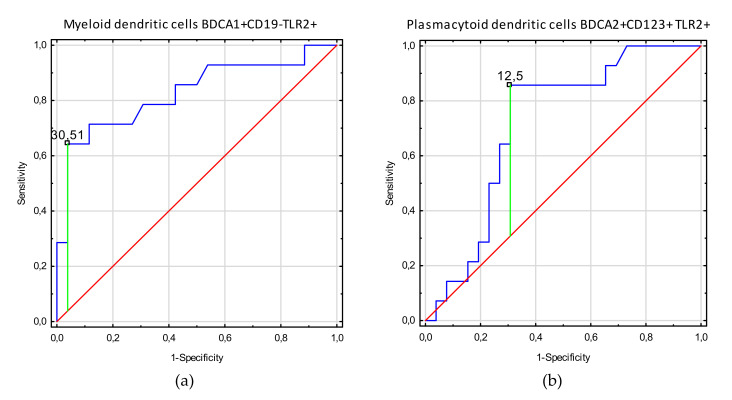

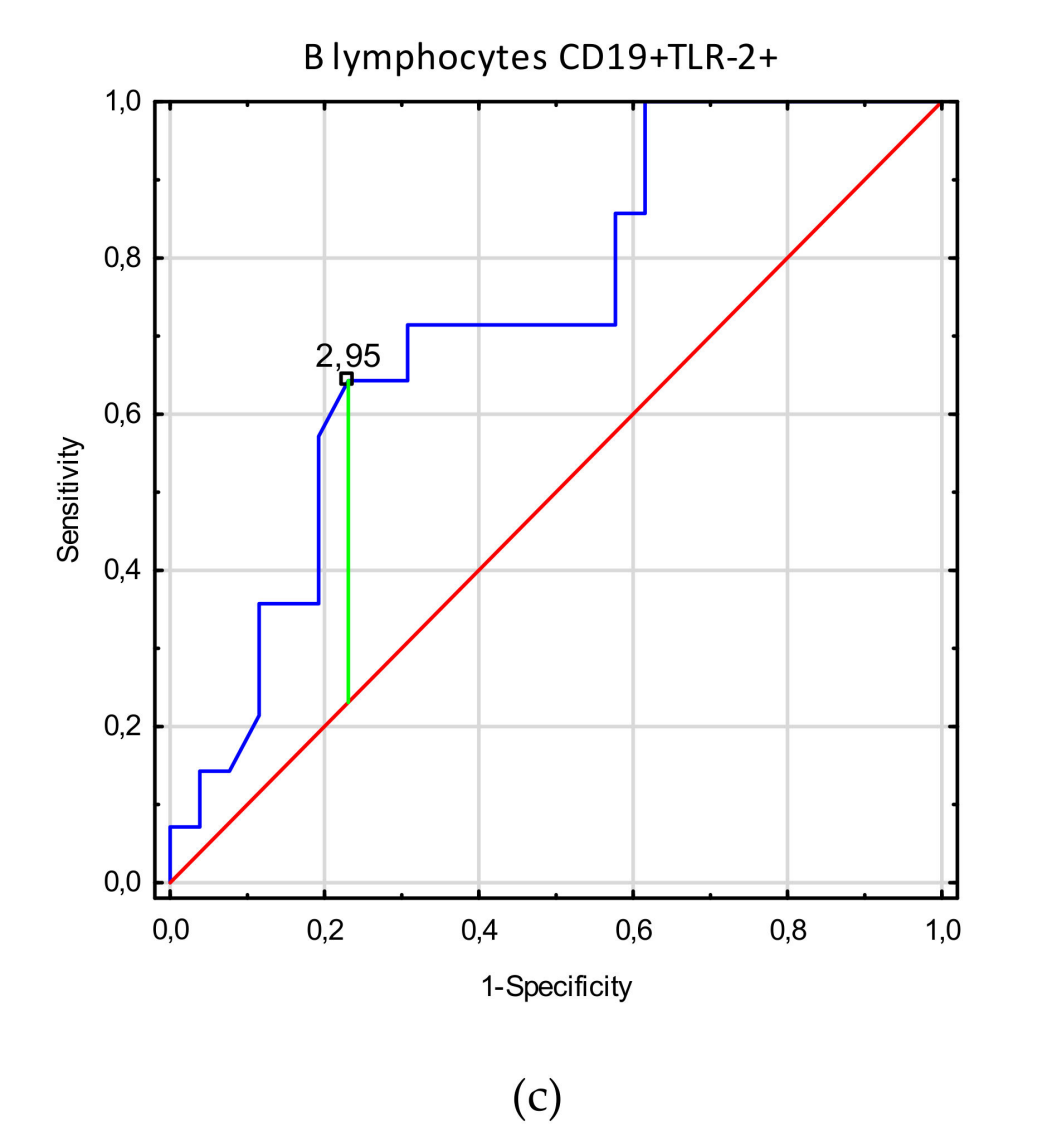
Receiver operating curve (ROC) analysis to determine diagnostic accuracy in differentiation of endometriosis patients with and without infertility; (**a**) frequencies of myeloid dendritic cells BDCA1+CD19-TLR2+ [%]; (**b**) frequencies of plasmacytoid dendritic cells BDCA2+CD123+ TLR2+ [%]; (**c**) frequencies of B lymphocytes CD19+TLR-2+ [%].

Table 4 shows the ROC analysis of clinical indices in endometriosis patients related to TLR2+ biomarkers. As the area under the curve (AUC) values show the increase of myeloid dendritic cell BDCA1+CD19-TLR2+ values for endometriosis patients in disease stages 3–4, with infertility and adhesion, this parameter render to be the most sensitive and specific parameter to determine who are endometriosis patients with these clinical symptoms. Diagnostic accuracy was excellent for B lymphocyte CD19+TLR-2+ parameters, which showed negative correlation with infertility, adhesion, and 3–4 stages in endometriosis patients. Moreover, good diagnostic accuracy was observed for plasmacytoid dendritic cell BDCA2+CD123+TLR2+ parameters showing negative correlation with infertility. Decrease of non-classical monocyte CD14+CD16+TLR2+ values below their prognostic value has good discriminative power between endometriosis patients with and without adhesion. Figure 2, Figure 3 and Figure 4 present biomarkers that demonstrated excellent and good diagnostic accuracy.

## 4. Discussion

### 4.1. The Amount of mDCs Increases Significantly in the Course of Endometriosis

Our study shows that myeloid DCs expressing TLR2 appear to be important in the pathophysiology of endometriosis, in view of their increased levels compared with the control group. Not only does the level of mDCs increase in affected patients, but also an association with clinical symptoms and stage of the disease was observed, which may allow for patient stratification. 

Such observations are the first in literature, while previously it was confirmed that in patients with endometriosis, immature DCs (iDCs) rather than mDCs are recruited [40]. Other studies show that the main subset in endometriosis is mDC type 1 with high expression of mannose receptor (MR), an important marker of successful phagocytosis [14]. Possibly, a dramatically high level of mDCs may also influence the presenting functions of these cells, leading to consequences visible in the adaptive immunity. 

### 4.2. Monocytes and Basic Peripheral Blood Lymphocytes and their Clinical Relation to Endometriosis

In our study, the levels of nonclassical monocytes CD14+CD16+TLR2+ in patients with endometriosis seem to have good discrimination power between endometriosis patients with and without adhesions.

Nevertheless, the results of the previous studies of monocytes/macrophages subsets in patients with endometriosis are inconsistent. It was shown that macrophages infiltrate endometriotic lesions [18], and the number of this cell population increases in patients with endometriosis [41]. In addition, the lack of peripheral monocytes and peritoneal macrophages keeps the capability to adhere to peritoneal layer [18]. The inhibition of phagocytosis ability of macrophages is also noted in patients with endometriosis, due to lower expression of CD36 caused by prostaglandin E2 [42], possibly playing a role in the development of the disease. Others factors, like estrogen receptor (ER) alpha, ERbeta, CD68, NCL-MACRO, and HAM56 and inflammatory cytokines IL-1beta, TNF-alpha, and IL-6, as well as pro-inflammatory transcription factor NF-κB are upregulated in macrophages from peritoneal fluid in patients with endometriosis [43,44]. High expression of CD163 and CD206, required in scavenging of hemoglobin with iron transfer into macrophages, is also registered in patients with endometriosis [18]. Moreover, an increase in secretion of protein MCP-1 in macrophages in cases of women with endometriosis influences the recruitment of these cells to sites of injury and inflammation [45]. 

Summing up, the decrease in monocyte count is, surprisingly, associated with lower adherence and reduced efficiency of phagocytosis. The cascade of functions exploited by monocytes/macrophages, not only in the early immune response, but also later in the cytokine production [8], may confirm an important role of these cells in the pathogenesis of endometriosis [8]. 

Interestingly, the level of T CD8+TLR2+ is markedly higher in the stages 3–4 of endometriosis than in stages 1–2, suggesting the potential mobilization of these cells in more advanced stages of the disease. We also observed a weak, but statistically significant correlation between T CD4+TLR2+ lymphocytes and HE4 serum concentration, which is a biomarker for ovarian cancer and until now was understood as a hormone-dependent factor [46]. No corresponding results were found in the literature. It was, however, observed that Th1 profile dominates over the Th2 responses in patients with endometriosis [8]. More diverse studies have been performed on the Tregs, which are present in endometriotic lesions [25,47], but interestingly no significant correlation was found between the Tregs and white blood cell count, suggesting that the local host defense mechanism is deficient in patients with endometriosis [47]. 

Our study also showed a negative association of B lymphocyte CD19+TLR-2+ levels with infertility, adhesion, and stages III and IV in patients with endometriosis. It can be speculated that this is related to the impaired presenting function of DCs. Hypothetically, this may be either due to asymptomatic infection or reduced reactiveness of the immune system. Such an observation is reported for the first time, while so far, only high serum levels of IgG, IgA, and IgM autoantibodies, and anti-endometrial antibodies [8] or no significant differences between the levels of B cells in patients with endometriosis [48] were observed. 

### 4.3. TLR2 Expression is Implicated in the Pathogenesis of Endometriosis 

Our study shows that TLR2 expression on the observed immune cells plays a role in endometriosis, not only for the dynamics of these cells’ presence in the course of endometriosis, but also in impacting disease manifestation. TLRs mediate both infection and sterile injury in response to microbial and endogenous factors. A comprehensive review of the literature concerning immunological and inflammatory factors possibly predisposing to endometriosis showed that, hypothetically, at least two phases can be distinguished in the development of this condition: the initial activation of TLR, which modulates innate immune response, followed by the phase of sterile inflammation [49]. Inflammation induced by a bacterial infection can trigger the continuous activation of TLRs, and thus, may contribute to the initialization of endometriosis development. As a result of further TLR stimulation by the second-wave oxidative stress, the inflammation may become persistent and can further exert antiapoptotic effects [49]. Decreased rates of apoptosis represent one of the primary mechanisms for pathogenesis of endometriosis [49,50]. The process of initializing chronic, aseptic inflammation, although still unclear, might be mediated by danger-associated molecular patterns (DAMPs) released from the damaged endometriotic lesions [49]. Several endogenous DAMPS, including DNA, high-mobility group box protein 1, adenosine-5′-triphosphate, and heat shock protein 70, were demonstrated to be associated with endometriosis [51].

Studies on TLRs in patients with endometriosis are very limited, with just a few data on the presence of TLR1, 2, 3, 4, 5, and 6 in the epithelia of different regions of the female reproductive tract [52]. One study also shows that TLR3 and TLR4 expression is decreased in patients with endometriosis [53]. TLR4 is present in the endocervix, endometrium, and uterine tubes and absent in vagina and ectocervix, and, according to Fazeli et al. [52], mainly TLR4 may play a crucial role in immunity of the female reproductive tract. The possible interconnection between bacterial infection, lipopolysaccharide, and TLR4 in the development of endometriosis was pointed out by Khan et al. [54]. The expression of TLR1-6 and the absence of TLR10 were confirmed in the endometrial biopsies by Schaefer et al. [55]. In this study, it was also shown [55] that TLR2 induces IL-6 and IL-8, which usually, together with IL-1α and TNF-α, elicits the recruitment and activation of macrophages, DCs, neutrophils, and lymphocytes to the site of infections, which also cannot be excluded in our study. 

Hypothetically, TLR2-mediated expression of DCs in patients with endometriosis is the trigger of the endogenous pattern of activation of TLRs by DAMPs [56]. This concept was introduced as an answer to redefining the immune system as the one that recognizes non-self from self, but rather the dangerous from safe [57]. Similarly, as in several inflammatory conditions, possibly in endometriosis, TLR2 reacts on an endogenous ligand to trigger DCs. It is still unknown which pattern of expression is undertaken in endometriosis. The upregulated levels of DAMP expression might be relevant for the process of sterile inflammation [49]. TLR2 expression seems to play an important role in endometriosis, potentially in the context of DAMP-mediated pathway. Nevertheless, further studies are undoubtedly needed to expand on the molecular character of this phenomenon. 

Our findings seem to be particularly relevant, because TLR2-expressing cells in the peripheral blood may be used in clinical practice as noninvasive biomarkers of the disease. Currently, although intensive research is being conducted, the clinical usability of endometriosis biomarkers, in particular those that are noninvasive, is scarce [3]. Given that most disease symptoms are unspecific, the availability of such biomarkers would be invaluable to allow for early and cost-effective diagnosis. Endometrial differences between healthy women and those affected by endometriosis were investigated in numerous studies to search for minimally invasive diagnostic options [4,5]. Among the most promising findings were those related to cell survival and apoptosis. One report highlighted the enhanced expression of pro-apoptotic pathways in endometriosis [58], while two others associated this condition with reduced apoptotic activity of endometrial cells [59,60]. Additionally, the presence of the nerve fibers in the endometrial biopsies appeared as an attractive putative biomarker of endometriosis, demonstrating specificity and sensitivity greater than 80% [61,62]. A review summarizing 25 years of research on peripheral biomarkers of endometriosis pointed out more than 100 candidates described in the literature [6], including the levels of serum CA-125 [63]. Nevertheless, their current clinical usefulness was assessed as inadequate [3]. Our study provides the rationale for further investigations on the potential of TLR2-expressing cells as promising peripheral biomarkers of the disease and for stratification of affected individuals.

## 5. Conclusions

Our study is the first to show the importance of TLR expression, mainly on mDCs, correlating with clinical output. Our findings also provide the rationale for the potential application of monitoring the level of TLR2-expressing mDCs as biomarkers of the disease and for the stratification of affected individuals. Further studies are needed in order to check the cause of this signaling pathway (endo or exogenous) and the localization of TLR2 expression, which can be done with planned histopathological studies. Currently, the lack of noninvasive biomarkers of endometriosis contributes to the main barriers to early diagnosis of the disease. Further studies on the clinical potential of TLR-expressing cells in the peripheral blood as endometriosis biomarkers are warranted as a step toward better disease management.

## Figures and Tables

**Table 1:** Basic characteristics of the study and the control groups.

Parameter	Endometriosis (n = 40)	Control Group (n = 40)	*p*-Value
	Mean ± SD/Median (Range)	Mean ± SD/Median (Range)	
Age [years]	35.5 ± 8.0	36.1 ± 10.7	NS
Ca-125 [U/mL]	31.8 (10.1–126.1)	13.5 (2.0–26.3)	<0.0001
HE4 [pmol/L]	38.7 (24.0–73.0)	32.6 (11.0–55.7)	0.025
Clinical features	N (%)	N (%)	
Stage of endometriosis:			N/A
I	10 (25)	N/A	
II	12 (30)	N/A	
III	7 (17.5)	N/A	
IV	11 (27.5)	N/A	
Adhesions	18 (45)	N/A	
Dysmenorrhea	26 (65)	N/A	
Infertility	14 (35)	N/A	

NS = not significant, N/A = not applicable, CA-125 = Cancer Antigen 125, HE4 = Human Epididymis Protein 4.

**Table 2:** The frequencies of the dendritic cells, monocytes, and basic peripheral blood lymphocyte subsets as well as the frequencies of these cell subsets expressing TLR2 antigen, for the patients and controls.

Parameter [%]	Endometriosis Group (n = 40)	Healthy Control Group (n = 40)	t/Z Value	*p*-Value
	Mean ± SD/Median (Range)	Mean ± SD/Median (Range)		
Myeloid Dendritic Cells BDCA1+CD19-	0.28 ± 0.2	0.41 ± 0.15	−3.20	0.0014
Plasmacytoid dendritic cells BDCA2+CD123+	0.32 ± 0.2	0.29 ± 0.17	0.39	0.69
Myeloid dendritic cells BDCA1+CD19−/Plasmacytoid dendritic cells BDCA2+CD123+ ratio	0.88 (0.04–5.0)	1.7 (0.31–6.3)	−3.13	0.0017
Classical monocytes CD14+CD16−	85.1 ± 5.5	90.9 ± 3.5	−4.76	<0.0001
Non classical monocytes CD14+CD16+	10.7 ± 4.9	5.3 ± 2.5	5.00	<0.0001
T lymphocytes CD3+	73.0 (61.9−78.8)	72.4 (61.6−79.7)	0.51	0.61
B lymphocytes CD19+	11.5 ± 3.2	11.7 ± 2.7	−0.48	0.63
NK cells CD3−CD16+CD56+	11.0 ± 4.2	14.7 ± 3.4	−3.80	0.0001
NKT-like cells CD3+CD16+CD56+	1.7 (0.24–11.3)	3.3 (1.2–4.9)	−1.66	0.096
T lymphocytes CD3+CD4+	40.2 (26.6–53.1)	40.3 (33.4–57.4)	−0.37	0.71
T lymphocytes CD3+CD8+	29.3 ± 5.5	30.2 ± 4.9	−1.20	0.23
T lymphocytes ratio CD3+CD4+/T CD3+CD8+	1.4 (0.73–2.7)	1.3 (0.93–4.5)	0.54	0.59
Myeloid dendritic cells BDCA1+CD19-TLR2+	21.3 (6.1–48.0)	3.5 (1.1–9.3)	7.45	<0.0001
Plasmacytoid dendritic cells BDCA2+CD123+ TLR2+	12.7 (2.5–43.9)	5.8 (0.98–14.3)	4.13	<0.0001
Classical monocytes CD14+CD16-TLR2+	8.3 ± 4.8	4.7 ± 2.1	3.37	0.0007
Non classical monocytes CD14+CD16+TLR2+	10.1 (1.5–45.6)	4.4 (0.66–11.6)	4.16	<0.0001
T lymphocytes CD4+TLR-2+	3.8 (0.47–40.1)	0.92 (0.04–4.1)	5.47	<0.0001
T lymphocytes CD8+TLR-2+	2.4 (0.1–59.4)	1.0 (0.08–6.5)	2.78	0.0054
B lymphocytes CD19+TLR-2+	4.2 (0.42–23.5)	2.6 (0.14–7.0)	2.67	0.0077

**Table 3:** The frequencies of the dendritic cell, monocyte, and basic peripheral blood lymphocyte subsets as well as the frequencies of these cell subsets expressing TLR2 antigen, for patients with endometriosis stage 1, 2, 3, and 4.

Parameter	Stage of the Disease (Mean + SD)				*p*-Value
	1	2	3	4	
Myeloid dendritic cells BDCA1+CD19-	0.26 ± 0.21	0.34 ± 0.26	0.21 ± 0.11	0.29 ± 0.17	0.80
Plasmacytoid dendritic cells BDCA2+CD123+	0.41 ± 0.21	0.21 ± 0.14	0.32 ± 0.2	0.36 ± 0.22	0.09
Myeloid dendritic cells BDCA1+CD19−/Plasmacytoid dendritic cells BDCA2+CD123+ ratio	0.80 ± 0.68	1.9 ± 1.5	0.73 ± 0.18	1.0 ± 0.5	0.13
Classical monocytes CD14+CD16-	87.4 ± 3.1	87.1 ± 5.1	83.6 ± 3.5	81.9 ± 7.0	0.064
Non-classical monocytes CD14+CD16+	8.6 ± 4.2	9.5 ± 5.0	10.8 ± 2.7	13.7 ± 5.5	0.09
T lymphocytes CD3+ [%]	71.5 ± 4.6	73.1 ± 2.8	69.6 ± 6.1	71.8 ± 2.9	0.47
B lymphocytes CD19+ [%]	12.2 ± 3.2	11.1 ± 2.9	12.7 ± 3.3	10.5 ± 3.4	0.37
NK cells CD3-CD16+CD56+ [%]	11.2±5.8	9.9±3.8	10.5 ± 4.6	12.4 ± 2.9	0.54
NKT-like cells CD3+CD16+CD56+ [%]	2.4±2.1	3.1±3.0	2.7 ± 2.7	4.1 ± 3.5	0.56
T lymphocytes CD3+CD4+ [%]	41.7±6.7	39.8±3.9	36.3 ± 3.2	42.6 ± 7.2	0.076
T lymphocytes CD3+CD8+ [%]	28.0 ± 4.7	30.8 ± 5.2	31.6 ± 7.7	27.2 ± 4.4	0.28
T lymphocyte ratio CD3+CD4+/T CD3+CD8+	1.6 ± 0.5	1.3 ± 0.37	1.2 ± 0.39	1.6 ± 0.5	0.21
Myeloid dendritic cells BDCA1+CD19−TLR2+	12.0 ± 3.7	18.6 ± 6.4	27.3 ± 3.7	33.7 ± 7.1	<0.0001
Plasmacytoid dendritic cells BDCA2+CD123+ TLR2+	12.2 ± 8.9	12.1 ± 7.0	19.2 ± 9.4	13.1 ± 11.2	0.19
Classical monocytes CD14+CD16-TLR2+	6.5 ± 3.7	7.7 ± 4.2	11.5 ± 6.5	8.6 ± 4.7	0.38
Non-classical monocytes CD14+CD16+TLR2+	8.3 ± 3.3	14.2 ± 7.5	14.4 ± 15.2	9.4 ± 6.0	0.21
T lymphocytes CD4+TLR-2+ [%]	8.5 ± 12.3	7.7 ± 4.8	5.6 ± 6.5	3.9 ± 3.2	0.36
T lymphocytes CD8+TLR-2+ [%]	1.7 ± 1.4	8.3 ± 7.5	11.4 ± 21.2	10.6 ± 18.0	0.10
B lymphocytes CD19+TLR-2+ [%]	13.0 ± 6.4	5.0 ± 3.9	3.9 ± 3.1	2.5 ± 1.8	0.0007

**Table 4:** Receiver operating characteristic (ROC) analysis to determine diagnostic accuracy in the differentiation of patients with endometriosis.

Factor	Parameter [%]	Prognostic Value	Youden Index	Area under the Curve (AUC)	95% CI	*p*-Value
Stages 3–4	Myeloid dendritic cells BDCA1+CD19-TLR2+	22.22	0.91	0.96	0.89–1.0	<0.0001
	B lymphocytes CD19+TLR-2+	5.62	0.49	0.78	0.64–0.95	0.0001
Adhesion	Myeloid dendritic cells BDCA1+CD19-TLR2+	20.45	0.81	0.92	0.83–1.0	<0.0001
	B lymphocytes CD19+TLR-2+	6.16	0.59	0.82	0.70–0.95	<0.0001
	Non classical monocytes CD14+CD16+TLR2+	10.0	0.39	0.70	0.53–0.87	0.018
Infertility	Myeloid dendritic cells BDCA1+CD19-TLR2+	30.51	0.60	0.83	0.68–0.98	<0.0001
	Plasmacytoid dendritic cells BDCA2+CD123+ TLR2+	12.5	0.55	0.71	0.55–0.88	0.009
	B lymphocytes CD19+TLR-2+	2.95	0.41	0.73	0.57–0.89	0.006

## Supplementary Materials

The following are available online at https://www.mdpi.com/2073-4409/9/8/1813/s1, Figure S1: Isotype controls: (**a**) Sample analysis of monocytes; (**b**) Sample analysis of myeloid dendritic cells; (**c**) Sample analysis of plasmacytoid dendritic cells; Figure S2: (**a**) Sample analysis of TLR-2+ monocytes; (**b**) Sample analysis of TLR-2+ myeloid dendritic cells; (**c**) Sample analysis of TLR-2+ plasmacytoid dendritic cells; Figure S3: Sample isotype controls for B CD19+ lymphocytes, T CD4+ lymphocytes and T CD8+ lymphocytes; Figure S4: Sample analysis of TLR-2+ B lymphocytes, T CD4+ lymphocytes and T CD8+ lymphocytes; Figure S5: Positive correlation between the frequencies of T CD4+TLR2+ lymphocytes [%] and HE4 serum concentration (*r* = 0.395; *p* = 0.0112).

## Abbreviations

BDCA	Blood Dendritic Cell Antigen
DC	Dendritic Cell
CD	Cluster of Differentiation
TLR	Toll-like receptor
