# Supplementary material for: Toll-Like Receptor 2 Expression as a New Hallmark of Advanced Endometriosis
Source: Cells. 2020 Jul 30;9(8):1813. doi: 10.3390/cells9081813 (PMC7464841; doi:10.3390/cells9081813)
Supplement: Supplementary file 1 [file cells-09-01813-s001.pdf]

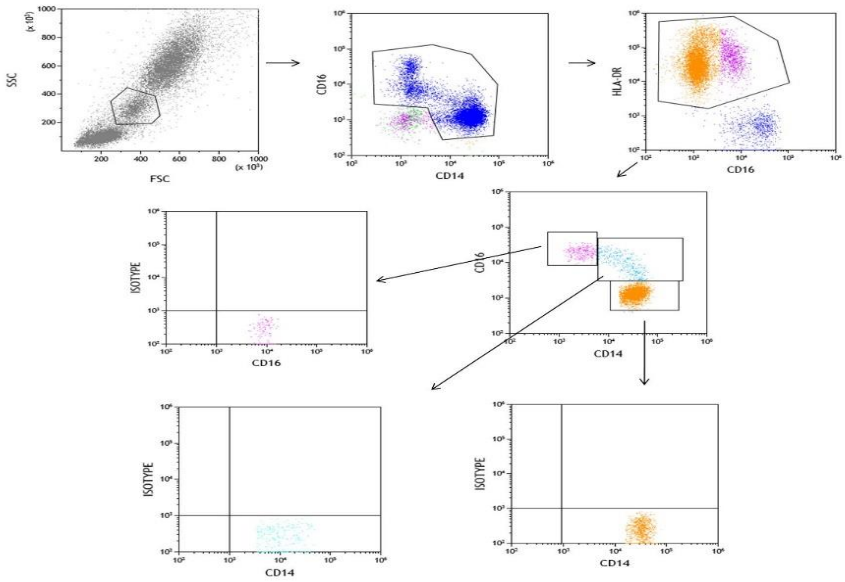

(a)

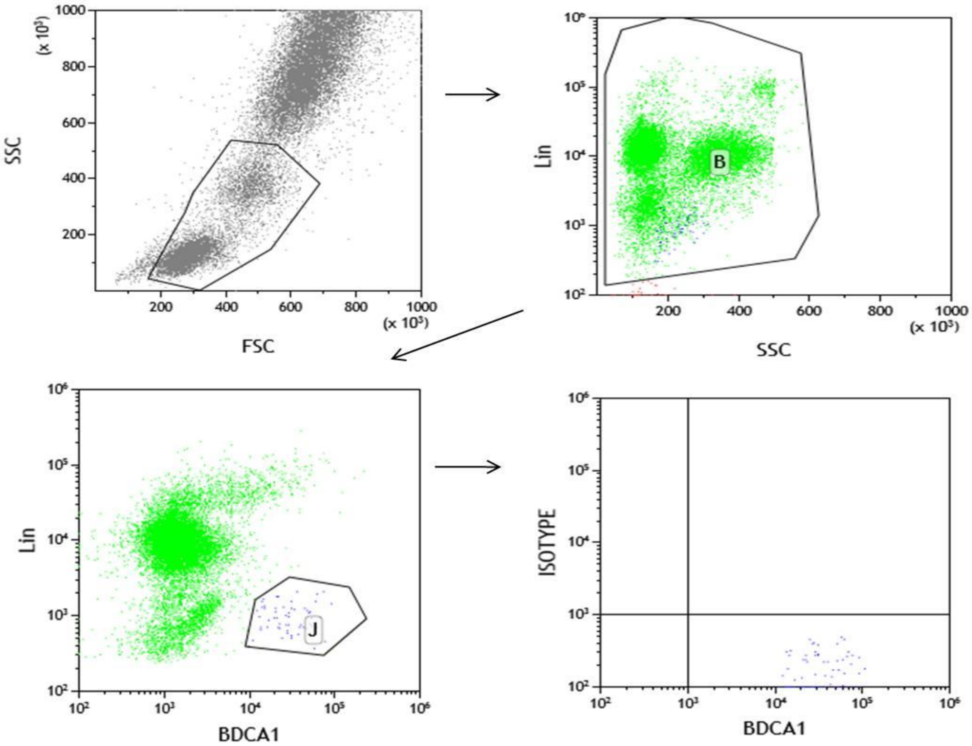

(b)

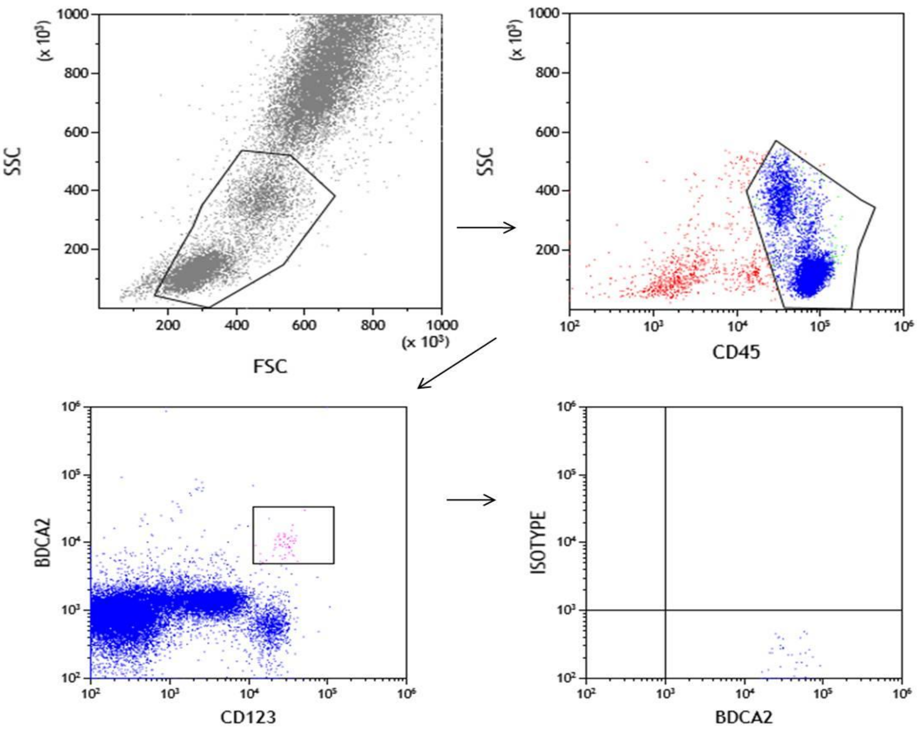

(c)

Figure S1. Isotype controls: (a) Sample analysis of monocytes; (b) Sample analysis of myeloid dendritic cells; (c) Sample analysis of plasmacytoid dendritic cells.

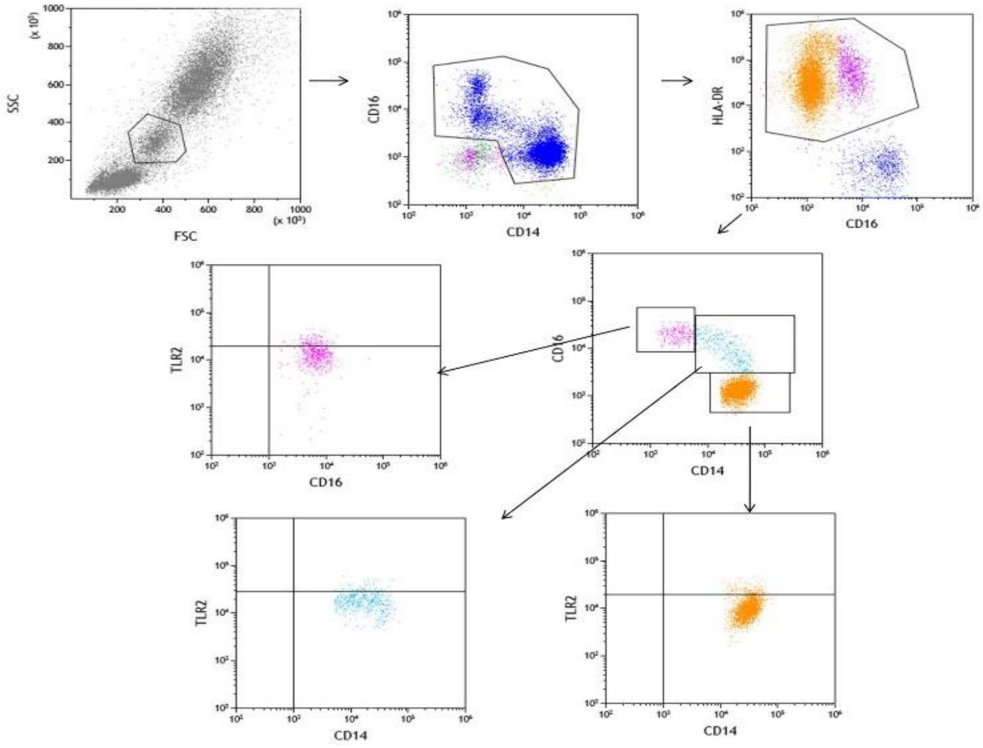

(a)

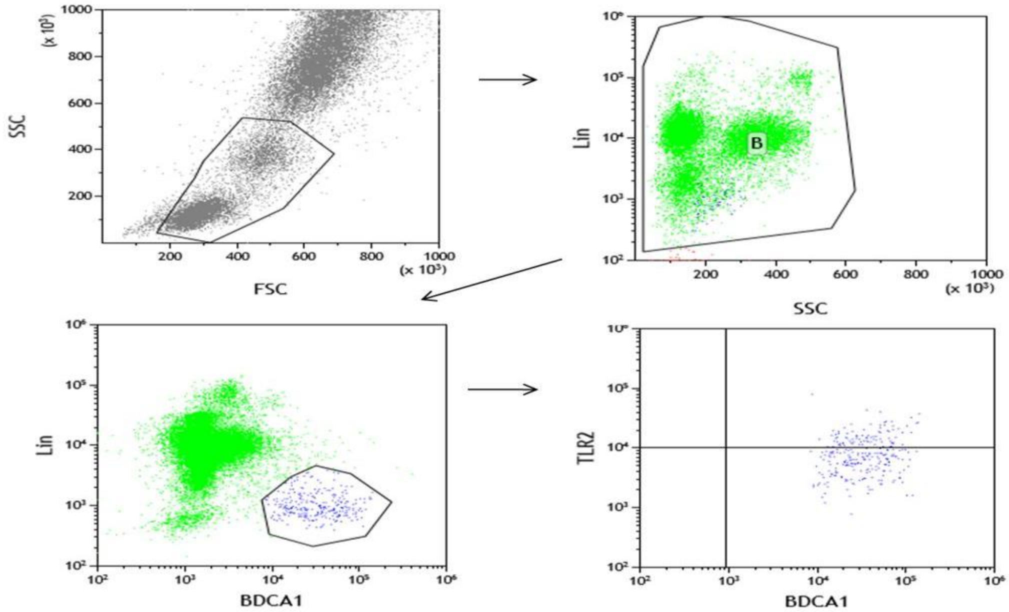

(b)

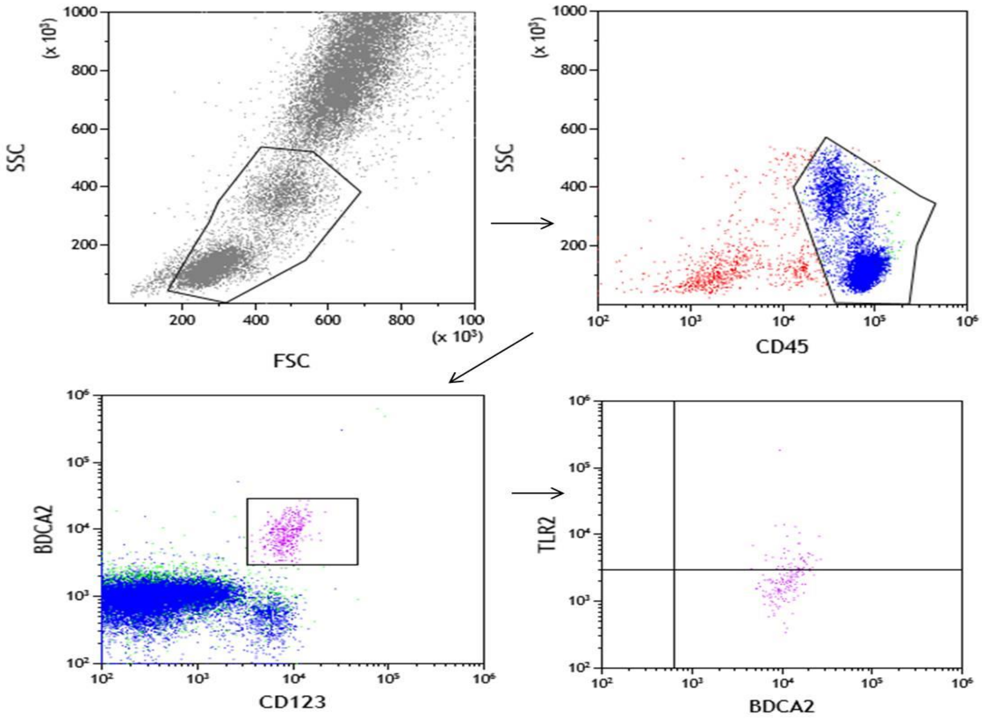

(c)

Figure S2. (a) Sample analysis of TLR-2+ monocytes; (b) Sample analysis of TLR-2+ myeloid dendritic cells; (c) Sample analysis of TLR-2+ plasmacytoid dendritic cells.

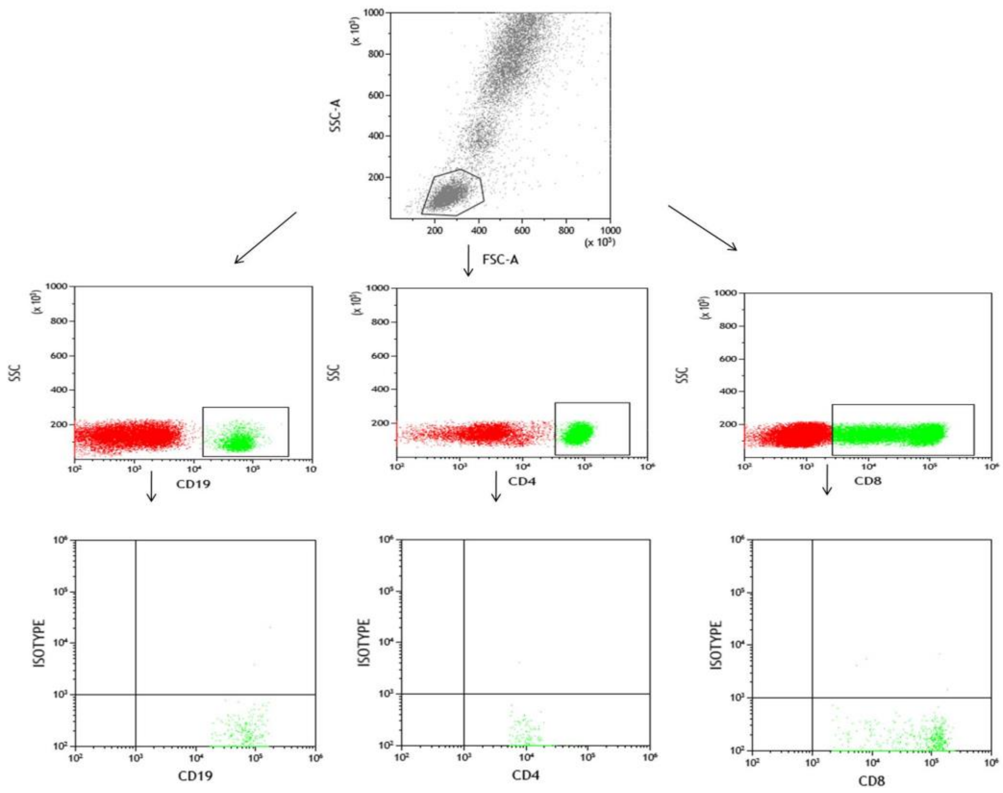

Figure S3. Sample isotype controls for B CD19+ lymphocytes, T CD4+ lymphocytes and T CD8+ lymphocytes.

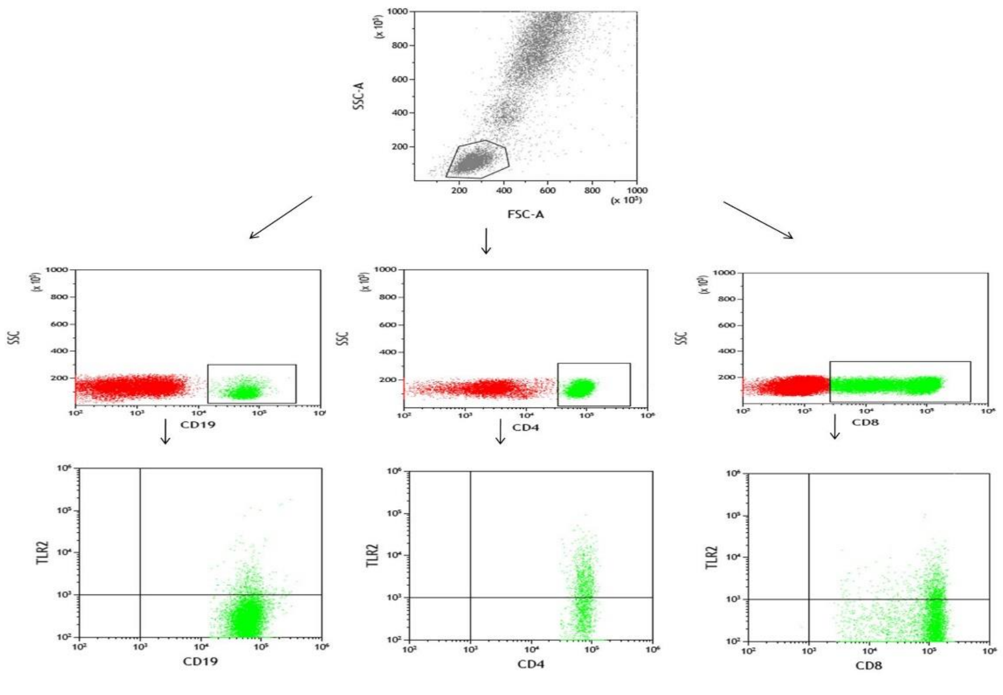

Figure S4. Sample analysis of TLR-2+ B lymphocytes, T CD4+ lymphocytes and T CD8+ lymphocytes.

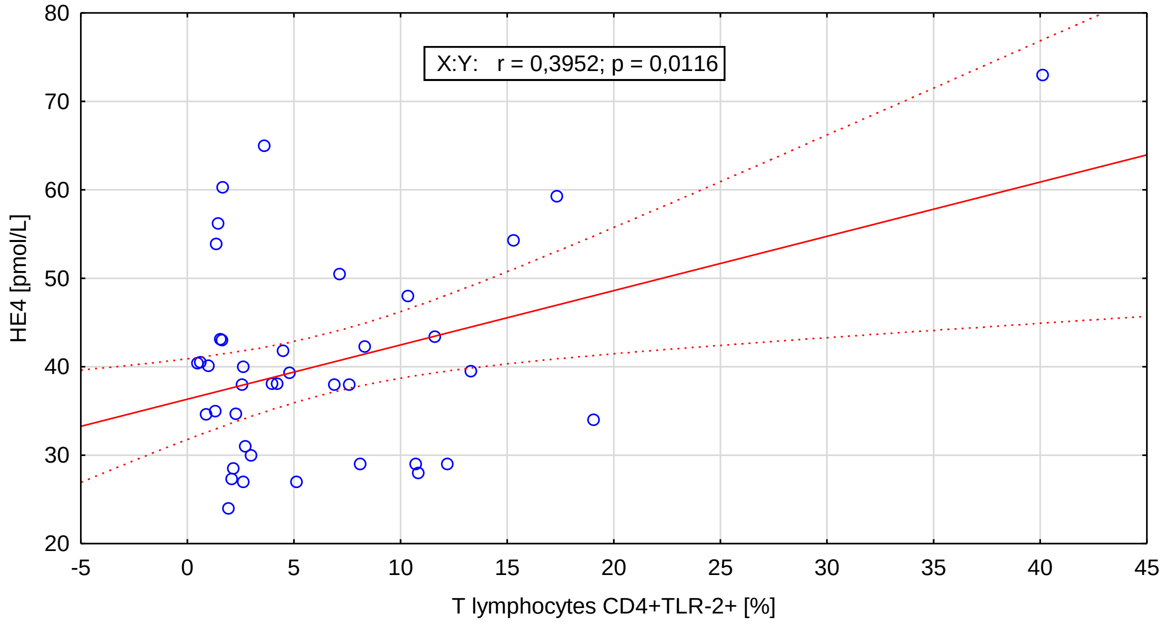

Figure S5. Positive correlation between the frequencies of T CD4+TLR2+ lymphocytes [%] and HE4 serum concentration ($r=0.395$; $p=0.0112$).
